# New Wild-Type *Lacticaseibacillus rhamnosus* Strains as Candidates to Manage Type 1 Diabetes

**DOI:** 10.3390/microorganisms10020272

**Published:** 2022-01-25

**Authors:** Grigorios Nelios, Valentini Santarmaki, Chrysoula Pavlatou, Dimitra Dimitrellou, Yiannis Kourkoutas

**Affiliations:** 1 Laboratory of Applied Microbiology and Biotechnology, Department of Molecular Biology & Genetics, Democritus University of Thrace, 68100 Alexandroupolis, Greece; gregnelios@hotmail.com (G.N.); vsantar@mbg.duth.gr (V.S.); cpavlato@mbg.duth.gr (C.P.); 2 Department of Food Science and Technology, Ionian University, 28100 Argostoli, Greece; dimitrellou@gmail.com

**Keywords:** *Lacticaseibacillus rhamnosus*, type 1 diabetes, probiotics, foodborne pathogens, *α*-glucosidase inhibitory activity

## Abstract

The incidence of type 1 diabetes (T1D) has been dramatically increased in developed countries, and beyond the genetic impact, environmental factors, including diet, seem to play an important role in the onset and development of the disease. In this vein, five *Lacticaseibacillus* *rhamnosus*, isolated from traditional fermented Greek products, were screened for potential probiotic properties, aiming at maintaining gut homeostasis and antidiabetic capability to alleviate T1D symptoms. *L. rhamnosus* cell-free supernatants induced strong growth inhibitory activity against common food spoilage and foodborne pathogenic microorganisms, associated with several diseases, including T1D, and were also able to inhibit *α*-glucosidase activity (up to 44.87%), a promising property for alternatives to the antidiabetic drugs. In addition, survival rates up to 36.76% were recorded during the application of the static in vitro digestion model. The strains had no hemolytic activity and were sensitive to common antibiotics suggested by the European Food and Safety Association, apart from chloramphenicol. However, it is highly unlikely that the resistance has been acquired. In conclusion, our results suggest a great health-promoting potential of the newly isolated wild-type *L. rhamnosus* strains, but further confirmation of their efficiency in experimental animal models is considered an essential next research step.

## 1. Introduction

Today, an upsurge of interest in developing novel functional foods containing probiotic microorganisms is witnessed. According to the World Health Organization and Food and Agriculture Organization (WHO/FAO), probiotics are commonly defined as viable microorganisms (bacteria or yeasts) that, when administered in adequate amounts, confer a health benefit on the host [1]. Several studies available in the literature have clearly shown that probiotics are effective in reducing the risk of antibiotic-associated diarrhea and its duration in healthy children [2,3], reducing levels of coliforms in different parts of the intestine [4,5,6], modulating gut microbiota and preventing sensitization to foods [7,8], improving glucose intolerance and immune responses [9,10,11,12], etc. 

To induce the health benefits, probiotics should be able to survive the acidic conditions of the upper gastrointestinal (GI) tract and proliferate and colonize in the gut [13,14], since they may influence interaction with the host and the other bacteria present, affect the local microbial composition, and/or stimulate the host’s immune system [12,15].

Type 1 diabetes (T1D) mellitus is characterized by the destruction of insulin-producing pancreatic beta cells, leading to insulin deficiency and increased glucose levels in blood and urine. Hyperglycemia in patients with T1D can be fatal if not treated with insulin, and some of its most recurrent clinical symptoms are weight loss, polyuria, polydipsia, and polyphagia [16]. In the last decades, the incidence of T1D has been dramatically increased in developed countries, and beyond the genetic impact, environmental factors, including diet, seem to play an important role in the onset and the development of the disease. The intestinal microbiome might affect the interaction between the GI tract and the immune system and result in altered immune responses, affecting the development of T1D [17]. Thus, the restoration of the normal microbiota composition, which could be accomplished with a probiotic-rich diet, constitutes a new target for the prevention and management of the disease. 

At the same time, food spoilage and safety are considered among the most pressing public health issues, due to economic reasons and constant outbreaks of food-borne diseases. As a result, probiotics, such as lactic acid bacteria (LAB), have recently received increased interest in the food industry for maintaining human health and acting as a shield against food spoilage microorganisms and pathogens. The antimicrobial activity of LAB, which has been studied against a variety of spoilage microorganisms and several healthcare-associated pathogens [18,19,20], has been attributed to a wide spectrum of antimicrobial compounds, such as organic acids, bacteriocins, hydrogen peroxide, diacetyl, and others [21].

Indeed, foodborne pathogens have been associated with several diseases, including T1D. Due to the hyperglycemic environment, a dysfunction of the immune system is usually observed in T1D patients, resulting in the development of several serious either healthcare-associated or community-based infections. Thus, diabetic patients may have a predisposition to infections caused by enteric pathogenslike *Escherichia coli*, *Salmonella* Enteritidis, *Listeria monocytogenes*, *Clostridium difficile*, etc., due to gastrointestinal dysmotility syndromes associated with diabetes [22,23]. Furthermore, diabetic patients are susceptible to invasive fungal infections, such as pulmonary aspergillosis [24]. 

However, the health-promoting effects resulting from probiotics seem to be strain-specific rather than characteristic of a specific species. Therefore, the discovery and isolation of health-promoting bacteria are considered of utmost importance to any commercialization strategy of probiotic products.

In the present study, *Lacticaseibacillus rhamnosus* (previously classified as *Lactobacillus rhamnosus*) strains, isolated from traditional fermented Greek products, were screened in vitro for potential probiotic properties, aiming at maintaining gut homeostasis and antidiabetic capability to alleviate T1D symptoms. Data supporting significant growth inhibition activity of cell-free supernatants (CFSs) against common food spoilage microbes and food-borne pathogens, α-glucosidase inhibitory activity, and resistance to common antibiotics are presented.

## 2. Materials and Methods 

### 2.1. Isolation of Lactic Acid Bacteria

Brine from naturally fermented Greek olives and olive fruits (*Olea europaea* var. *rotunda*) collected at the end of the fermentation process, as well as homemade cheese samples produced with no starter cultures, were collected for isolation of lactic acid bacteria. In brief, olives (10 g), cheese (10 g), and brine (1 mL) samples were macerated into de Man, Rogosa, and Sharpe (MRS) broth medium (Condalab, Madrid, Spain) and incubated at 37 °C for 24 h. Τhereafter, decimal dilutions were prepared in sterile quarter-strength Ringer’s solution, and 1 mL of each appropriate dilution was poured onto MRS agar (Condalab, Madrid, Spain) medium and incubated at 37 °C for 48 h. Colonies with typical characteristics were collected from the highest dilution and were picked for routinely streaking to obtain pure colonies. Each strain was maintained at −80 °C in MRS broth/glycerol (70:30).

### 2.2. Bacterial Identification

All isolates were tested for Gram staining, cell morphology, and catalase reaction, and only Gram-positive and catalase-negative strains were collected. Initial characterization was performed by the API50CH biochemical system (BioMèrieux, La Balme-les-Grottes, France), according to the manufacturer’s instructions and using the APIweb^TM^ software. For molecular identification, the strains were inoculated into MRS broth and 1 mL of overnight culture was collected for genomic DNA extraction utilizing the Nucleospin tissue kit (Macherey-Nagel, Düren, Germany), according to the manufacturer’s instructions. Species-specific PCR reactions for the *Lacticaseibacillus casei* group were carried out in a volume of 20 μL with primers targeting *mutL* gene, i.e., CZfor (5′-CAGCGCTGGTGGAAGACTTG-3′), PC2a (5′-GGATTGGGTTTTGCGTGATGGTCGC-3′), RHfor (5′-GACTTCTCAACCAGCAGCGCAGA-3′), and CPRrev (5′-TGCATTTCCCCGCTTTCATGACT-3′), as described recently [25]. Amplification reactions were performed using an Eppendorf 5333 MasterCycler Thermal Cycler (Hamburg, Germany). PCR products were analyzed by electrophoresis (Bio-Rad Laboratories, Hercules CA, USA) in 1.5% agarose gel in 1% Tris-Borate-EDTA (TBE) and were visualized by UV transillumination (Bio-Rad Laboratories, Hercules, CA, USA).

### 2.3. Cell-Free Supernatant Preparation

Wild-type *L. rhamnosus* cultures were grown in MRS broth (pH 6.5) for 24 h at 37 °C, and then cell-free supernatants (CFSs) were obtained by centrifugation (8000× *g* for 20 min, 4 °C). CFSs were sterilized by filtration with a 0.22 μm filter (Merck, Darmstadt, Germany). *L. rhamnosus* GG (ATCC 53103) (Probiotical SpA, Novara, Italy) was used as a reference strain for comparison reasons.

### 2.4. Antimicrobial Activity of L. rhamnosus CFSs

#### 2.4.1. Food Spoilage and Pathogenic Microbial Strains

*Salmonella enterica* subsp. *enterica* ser. Enteritidis FMCC B56 PT4 (kindly provided by the Laboratory of Microbiology and Biotechnology, Agricultural University of Athens, Athens, Greece), *S. enterica* subsp. *enterica* ser. Typhimurium B62 DSM 554, *Escherichia coli* ATCC 25922 (kindly provided by Dr. Nisiotou A., Athens Wine Institute, ELGO-DIMITRA, Athens, Greece), *Listeria monocytogenes* NCTC 10527 serotype 4b, *Staphylococcus aureus* ATCC 25923, and *Clostridium difficile* (kindly provided by the Laboratory of Clinical Microbiology, Sismanoglio General Hospital, Athens, Greece) were grown in brain heart infusion (BHI) broth (LAB M, Heywood, UK) at 37 °C for 24 h, except *C. difficile*, which was incubated at 37 °C for 48 h. *Saccharomyces cerevisiae* Uvaferm NEM (Lallemand, Montreal, QC, Canada) was grown in yeast extract peptone dextrose (YPD) broth (yeast extract 10 g/L, glucose 20 g/L, and peptone 20 g/L) at 28 °C for 24 h. *Aspergillus niger* 19111 (kindly provided by G.J.E. Nychas, Agricultural University of Athens, Athens, Greece) was grown on Malt agar (Condalab, Madrid, Spain) at 37 °C for 7 days.

#### 2.4.2. Assessment of Growth Inhibition Activity of *L. rhamnosus* CFS

Growth inhibition activity against common food spoilage and pathogenic microorganisms was evaluated by the broth microdilution method, as described by Mayrhofer et al. [26], with slight modifications. Growth inhibition activity of various concentrations (3.12, 6.25, 12.5, 25, 50, 70, and 90%) of non-neutralized and neutralized (pH adjusted to 7.0 using 5 M NaOH) CFSs diluted to MRS broth was tested in a 96-well microplate. In each well, 100 μL of bacterial or yeast or fungus culture (10^5^ cfu/mL or 10^5^ spores/mL) and 100 μL of CFS were added to achieve a total volume of 200 μL [27], followed by incubation at 37 °C for 24 h, apart from *C. difficile*, which was incubated at 37 °C for 48 h, and *S. cerevisiae* Uvaferm NEM, which was incubated at 28 °C for 24 h. Wells with bacterial or yeast/fungal cultures with MRS broth containing no CFSs served as growth controls. The growth of spoilage microorganisms was monitored by measuring optical density (OD 620 nm) using VarioskanTM LUX Multimode Microplate Reader (Thermo Scientific, Vantaa, Finland). The percentage of growth inhibition was calculated based on the following equation:

$$I\left(\%\right)=\left(\frac{A620\left(\mathrm{control}\right)-A620\left(\mathrm{sample}\right)}{A620\left(\mathrm{control}\right)}\right)\times 100\%,$$

where I (%) is the percentage of growth inhibition of the sample compared with the growth of the control, A620 (control) represents the changes in the optical density during growth of the control, and A620 (sample) represents the changes in the optical density of the sample [19]. 

### 2.5. Determination of α-Glucosidase Inhibition 

α-Glucosidase inhibitory activity was assessed according to the method described by Zeng et al. [28], modified as follows. The reaction mixture containing 50 μL of CFS (pH adjusted to 7.4 with the addition of 5 M NaOH) or 10 mg/mL acarbose (reference inhibitor) (Sigma-Aldrich, St. Louis, MO, USA), 125 μL of phosphate-buffered saline (PBS) (0.80% NaCl, 0.02% KCl, 0.02% KH_2_PO_4_, and 0.22% Na_2_HPO_4_ (pH 7.4)), and 50 μL of 0.1 U/mL α-glucosidase from *Saccharomyces cerevisiae* (Type 1, ≥10 U/mg) (Sigma-Aldrich, St. Louis, MO, USA) was pre-incubated in a 96-well plate at 37 °C for 10 min. After pre-incubation, the reaction was initiated by adding 25 μL of 3 mM *p*-nitrophenyl α-D-glucopyranoside (*p*NPG) (Sigma-Aldrich, St. Louis, MO, USA), and incubation at 37 °C for 10 min was followed. Absorbance readings were recorded at 405 nm by VarioskanTM LUX Multimode Microplate Reader (Thermo Scientific, Vantaa, Finland), and α-glucosidase inhibition was calculated using the following equation:

$$\mathrm{CFS}\;\alpha -\mathrm{glucosidase}\;\mathrm{inhibition}\;\left(\%\right)=\left(1-\frac{A405\left(\mathrm{sample}\right)\;-\;A405\left(\mathrm{sample}\;\mathrm{blank}\right)}{A405\left(\mathrm{positive}\;\mathrm{control}\right)-A405\left(\mathrm{negative}\;\mathrm{control}\right)}\right)\times 100\%,$$

where A405 (sample) is the absorbance with the sample and α-glucosidase, A405 (sample blank) is the absorbance with the sample without α-glucosidase, A405 (positive control) is the absorbance with α-glucosidase without the sample, and A405 (negative control) is the absorbance without α-glucosidase or the sample. 

### 2.6. Static In Vitro Digestion Model

A static in vitro digestion model for probiotic strains screening was developed based on the previous study of Minekus et al. [29] with adaptations by Madureira et al. [30]. *L. rhamnosus* cells, grown in MRS broth, were harvested by centrifugation, washed twice with quarter-strength Ringer’s solution (Sigma-Aldrich, St. Louis, MO, USA), resuspended in simulated salivary fluid (SSF) [29] containing 75 U/mL α-amylase from *Bacillus* spp. (Type-IIA, ≥1500 U/mg) (Sigma-Aldrich, St. Louis, MO, USA) and 50 U/mL lysozyme from chicken egg white (≥20,000 U/mg) (Apollo Scientific, Cheshire, UK) and incubated at 37 °C for 2 min. Subsequently, cells were harvested once again by centrifugation, resuspended in simulated gastric fluid (SGF) [29] containing 2000 U/mL pepsin from porcine mucosa (≥3.200 U/mg) (Sigma-Aldrich, St. Louis, MO, USA) and incubated at 37 °C for 110 min. During incubation, pH in SGF was gradually reduced from 4.9 to 3.0 (10 min pH 4.5, 10 min pH 4.0, and 90 min pH 3.0), mimicking the transit from the oral cavity to stomach [30]. After the simulated gastric phase, cells were harvested by centrifugation again, resuspended in simulated intestinal fluid (SIF) [29] containing 100 U/mL pancreatin from porcine pancreas (8 × USP specifications) (Sigma-Aldrich, St. Louis, MO, USA) and incubated at 37 °C for 120 min. In all cases during the three simulated digestion phases, centrifugation conditions were 8000× *g*, 4 °C, 15 min, the harvested cells were resuspended in equal volumes, enzyme solutions were prepared fresh daily, and pH was adjusted with the addition of 0.5 M HCl or 1 M NaHCO_3_. Samples of 1 mL were collected right after the addition of SSF and after the end of each simulated digestion phase. Viable *L. rhamnosus* cell counts were determined by 10-fold serial dilution and pour plate method on MRS agar. MRS plates were incubated anaerobically at 37 °C for at least 72 h. The survival rates were calculated in accordance with the following equation [28]:

$$\mathrm{Survival}\;\mathrm{rate}\;\left(\%\right)=\frac{\mathrm{logcfu}\;\alpha }{\mathrm{logcfu}\;\beta }\times 100\%,$$

where α is the total count of viable cells after incubation for the specified digestion phase, and β is the total count of viable cells before the beginning of simulated digestion. 

### 2.7. Safety Evaluation

Hemolytic activity of isolates was examined on blood agar plates containing 5% (*w*/*v*) sheep blood. Fresh overnight cultures were standardized to 10^8^ cfu/mL and streaked on the plates. Colonies were investigated for surrounding zones (α and β hemolysis) after incubation at 30 °C for 48 h. *Staphylococcus aureus* MRSA, a clinical isolate kindly provided by the Laboratory of Clinical Microbiology, Sismanoglio General Hospital, Greece, was used as the positive control strain for β-hemolysis and *Lacticaseibacillus rhamnosus* GG as the negative control.

Furthermore, susceptibility to common antibiotics was also determined. The concentration ranges (mg/L) of the tested antibiotics were: ampicillin (0.032–16), clindamycin (0.032–16), erythromycin (0.016–8), gentamicin (0.5–256), streptomycin (0.5–256), tetracycline (0.125–64), kanamycin (2–1024), and chloramphenicol (0.125–64). Antibiotic susceptibility tests were performed using the broth microdilution method, according to the standard procedure of the International Organization for Standardization (ISO 10932/IDF 223:2010). Briefly, the inocula of the strains were prepared by suspending colonies in 0.85% NaCl solution and subsequently diluted in LAB susceptibility test medium (LSM) broth (HiMedia Laboratories, Mumbai, India). To each well, 100 μL of diluted inoculum and 100 μL of antibiotic solution were added, following incubation at 37 °C for 48 h. The minimum inhibitory concentration (MIC) was defined as the lowest concentration of antibiotics that inhibit the visible growth of the isolates, and the resulting values were compared with the microbiological cut-off values defined by the European Food Safety Authority’s (EFSA) recommendations [31].

### 2.8. Statistical Analysis

All treatments were carried out at least in duplicate. Statistical significance was determined by factorial analysis of variance (ANOVA), followed by Duncan’s multiple range test. Statistica version 10.0 was used to compute statistical significance at *p* < 0.05, coefficients, and ANOVA.

## 3. Results and Discussion

Probiotic bacteria should fulfill several requirements related to their ability in inhibiting the growth of food-spoilage microbes and pathogens, reducing α-glucosidase activity, reaching the lower GI tract intact, and being susceptible to antibiotics, among others.

### 3.1. L. rhamnosus Strains Isolation and Identification

**Figure 1:**
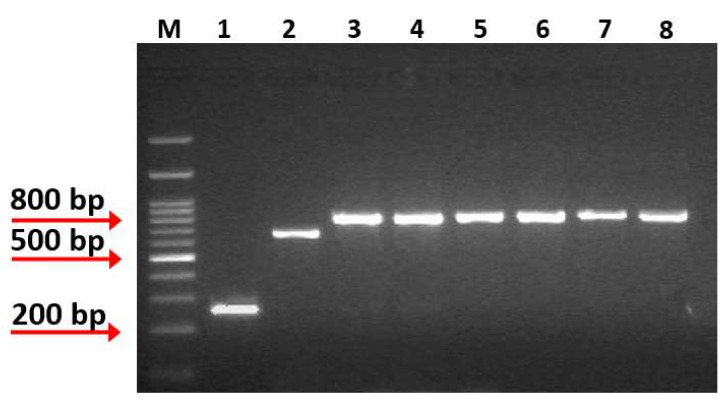
Multiplex PCR amplification products obtained from species-specific *mutL*-targeting primers assay. Lane M: 1kb DNA Ladder (Takara, Shiga, Japan), Lane 1: PCR product from *L. paracasei* DSM 20006, Lane 2: PCR product from *L. casei* ATCC 393, Lane 3: PCR product from *L. rhamnosus* GG, Lane 4: PCR product from isolate OLXAL-1, Lane 5: PCR product from isolate OLXAL-2, Lane 6: PCR product from isolate OLXAL-3, Lane 7: PCR product from isolate OLXAL-4, Lane 8: PCR product from isolate CHTH-2.

Strains isolated from brine, fermented table olives, and cheese samples were examined for catalase reaction, Gram staining, and stable colony morphology after subculturing on MRS agar, and five strains were collected for further analysis. These strains were non-motile, Gram-positive, catalase-negative, rod-shaped and were preliminarily identified as *Lacticaseibacillus rhamnosus* using API50CH biochemical tests. Further molecular confirmation was based on species-specific primers for the *Lacticaseibacillus casei* group and utilizing *Lacticaseibacillus rhamnosus* GG, *Lacticasebacillus casei* ATCC 393, and *Lacticaseibacillus paracasei* DSM 20006 as reference strains. Targeting the *mutL* locus revealed genome diversity among the *L. casei* group and effectively differentiated the species of *L. rhamnosus* (801 bp PCR product), *L. casei* (666 bp PCR product), and *L. paracasei* (253 bp PCR product) [25]. PCR products of 800 bp confirmed the initial characterization of the new isolates as *L. rhamnosus* (Figure 1). Of note, the species *L. rhamnosus* is included in the Qualified Presumption of Safety (QPS), a list indicating its suitability to food industry applications [32].

### 3.2. Growth Inhibition Activity of L. rhamnosus CFSs against Food Spoilage and Pathogenic Microorganisms

In the present study, we investigated the antagonistic activity of five *L. rhamnosus* strains against common food spoilage and pathogenic microorganisms. Food spoilage and pathogenicity are mainly due to the growth and activity of several microbial species, including, among others, *Escherichia coli*, *Salmonella* spp., *Listeria monocytogenes*, *Clostridium* spp., *Aspergillus* spp., and *Saccharomyces* spp. [33,34,35]. Infections by enteric pathogens are also usual in diabetic patients. For example, it has been previously reported that insulin may stimulate *E. coli* growth and enhance its ability to form biofilms [36]. Considering healthcare-associated infections, *C. difficile* has been related to high-risk rates in T1D patients [37,38], as they usually need to be hospitalized for a long time, increasing the risk of nosocomial infections. 

The CFSs (pH 3.72–3.87) induced strong growth inhibitory activity at high concentrations (≥ 25%) against all bacterial pathogens tested, reaching values up to 90–99.9% (Pavlatou, C. Laboratory of Applied Microbiology and Biotechnology, Department of Molecular Biology & Genetics, Democritus University of Thrace, Alexandroupolis, Greece. Growth Inhibition Activity of *L. rhamnosus* CFSs against Food Spoilage and Pathogenic Microorganisms, 2022). Reducing the concentration of CFSs down to 12.5%, the majority of CFSs maintained strong inhibitory activity (88–98%) against *S.* Enteritidis, *S.* Typhimurium, *E. coli*, *L. monocytogenes* and *S. aureus* (Figure S1). Regarding *C. difficile*, a significant (*p* < 0.05) reduction in the growth inhibitory ability of all CFSs at 12.5% concentration was observed (38.1–56.8%) compared with higher concentrations (25 and 50%) (Figure S1). At concentrations below 6.25%, all CFSs exhibited significantly (*p* < 0.05) lower growth inhibitory activity in all pathogens examined (Figure S1), in accordance with Chen et al. [27] and Arena et al. [39]. The only exception noted concerned CFSs of *L. rhamnosus* OLXAL-1 and *L. rhamnosus* OLXAL-2 that strongly inhibited the growth of *S. aureus* (69.7% and 67.9%, respectively) even at 3.12% concentration. Similarly, Inturri et al. [40] reported very strong inhibitory activity of CFSs at concentrations under 12.5% against *S. aureus*. 

Likewise, all CFSs exhibited strong growth inhibitory activity (88–100%) against *S. cerevisiae* and *A. niger* at 90% concentration. At lower concentrations (70%), a weaker (*p* < 0.05) growth inhibitory activity was observed (75–86% against *S. cerevisiae* and 65–79% against *A. niger*). Further decrease in CFSs concentration down to 50% resulted in even lower (*p* < 0.05) growth inhibition rates (55–63% against *S. cerevisiae* and 53–58% against *A. niger*) compared with higher concentrations (70% and 90%), while at CFSs concentrations below 25% a further growth inhibition (*p* < 0.05) was witnessed. 

**Figure 2:**
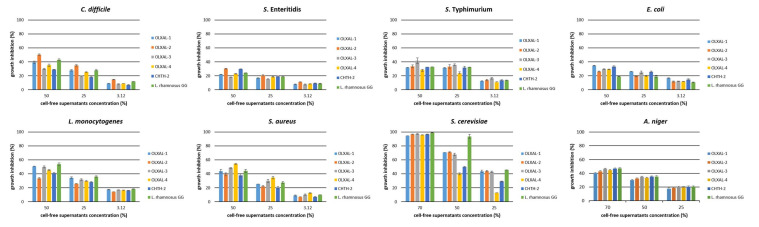
Growth inhibition activity of neutralized cell-free supernatants (CFSs) of *L. rhamnosus* strains against food spoilage and pathogenic microorganisms. Values are expressed as a percentage of growth inhibition compared with the control.

To investigate the nature of antimicrobial substances secreted by *L. rhamnosus* strains, the CFSs were submitted to neutralization and tested again as growth inhibitors. Neutralization of CFSs resulted in a drastic reduction (*p* < 0.05) in growth inhibitory activity against all bacterial pathogens tested (Figure 2), in accordance with Hor et al. [19] and Munoz et al. [41]. At 50% of neutralized CFSs concentration, the highest growth inhibitory activity was observed against *L. monocytogenes* and *S. aureus* (53.8% and 54.3%, respectively) (Figure 2). Lower concentrations (3.12, 6.25, 12.5, 25%) resulted in weaker (*p* < 0.05) antagonistic ability, reaching rates <36.2%. 

Similar results were also observed for *A. niger.* In specific, at concentration 50% and 70% of neutralized CFSs, 30–35% and 40–47% inhibitory activity, respectively, was observed, and at lower concentrations, antifungal activity was reduced, in accordance with previous studies [42,43].

On the other hand, all neutralized CFSs at 90% concentration maintained strong growth inhibitory activity (97.3–99.4%) against *S. cerevisiae* Uvaferm NEM (Pavlatou, C. Laboratory of Applied Microbiology and Biotechnology, Department of Molecular Biology & Genetics, Democritus University of Thrace, Alexandroupolis, Greece. Growth Inhibition Activity of *L. rhamnosus* CFSs against Food Spoilage and Pathogenic Microorganisms, 2022), similar to untreated CFSs. Interestingly, at 70% CFSs concentration, the inhibitory activity of neutralized CFSs remained strong (*p* > 0.05), in contrast with untreated CFSs, where the antagonistic activity was reduced (*p* < 0.05), probably due to the increase in pH, as optimum pH range for growth is 4 to 4.54 [44]. Further 2-fold dilutions of neutralized CFSs resulted in lower inhibition rates, and the highest values were observed in *L. rhamnosus* GG, used as reference strain (*p* < 0.05). 

The weaker growth inhibitory activity of neutralized CFSs against spoilage bacteria could be attributed to the organic acids resulting from cell metabolism. Production of organic acids leads to pH reduction, unsuitable for the growth of a wide spectrum of both Gram-positive and Gram-negative bacteria. More specifically, organic acids can pass through the cytoplasmic membrane of target microorganisms in their undissociated form, causing intracellular acidification and the collapse of the transmembrane proton motive force [39].

### 3.3. α-Glucosidase Inhibition

Insulin resistance is common among patients with T1D because of the constantly increasing doses of insulin, which in many cases results in weight gain. According to previous studies, concurrent administration of insulin and antidiabetic drugs, approved for the treatment of type 2 diabetes (T2D), may result in a reduction in insulin resistance and hence better weight management for individuals with T1D [45]. In particular, competitive inhibitors of α-glucosidase (miglitol and acarbose) have resulted in significant decreases in body mass index (BMI), total daily insulin dosages, hemoglobin A1c (HbA1c) and postprandial glucose levels [46,47,48]. However, in many cases, side effects related to the GI system, such as diarrhea and flatulence, have been reported [49,50,51]. In an attempt to avoid these adverse effects, oral supplementation of probiotic strains with α-glucosidase inhibitory activity is considered a promising alternative to the antidiabetic drugs [49,52,53]. In this vein, the α-glucosidase inhibitory activity of five wild-type *L. rhamnosus* strains was evaluated.

CFSs of all strains were able to inhibit α-glucosidase activity (Table 1). The inhibitory activities ranged from 35.43% to 44.87%, with the highest value recorded in CFS of *L. rhamnosus* OLXAL-1 strain (44.87%), significantly higher (*p* < 0.05) compared with *L. rhamnosus* GG (39.68%), which was used as a reference strain with well-documented antidiabetic properties [49]. According to previous literature, the α-glucosidase inhibitory activity of reference strain *L. rhamnosus* GG has been reported to vary between 13.5% and 37.9% [28,49,52,53], a result that leads to confusion concerning the comparability among different studies of the assay. This result disagreement probably stems from the several modifications implemented on each assay, such as the different origin of enzyme and varying concentrations of enzyme and substrate [54]. Nevertheless, in all the aforementioned studies, CFS of LAB strains exhibited α-glucosidase inhibition, which may be attributed to either secreted polysaccharides [52,55] or peptides produced from bacteria [28,56].

### 3.4. L. rhamnosus Cell Survival during In Vitro Digestion

Tolerance to gastric acidity, bile salts, and digestive enzymes consists an essential prerequisite for in vitro characterization of probiotics. However, in many cases, tolerance of probiotic strains to the harsh conditions of the GI tract is evaluated by separated static in vitro assays, which fail to simulate the successive stress conditions that occur in vivo [57,58]. Thus, the implementation of an in vitro digestion assay that incorporates oral, gastric, and intestinal phases consecutively can be a more efficient way to study the survival of presumptive probiotic strains. 

According to our results (Table 2), incubation in the simulated oral phase did not affect the initial viable cell counts of all *L. rhamnosus* strains tested, while incubation in the simulated gastric and intestinal phase led to a significant (*p* < 0.05) reduction. The survival rate in the simulated gastric phase ranged from 73.26% to 77.12%, indicating high tolerance to the acidic conditions for all strains. Simulated intestinal phase resulted in survival rates from 0 to 36.76%, while the reference strain *L. rhamnosus* GG showed zero survival rate, in accordance with Zeng et al. [28]. Overall, strains *L. rhamnosus* OLXAL-1, *L. rhamnosus* OLXAL-2, *L. rhamnosus* OLXAL-3, and *L. rhamnosus* OLXAL-4 demonstrated a survival rate >33.07% after simulated digestion, while *L. rhamnosus* CHTH-2 showed a significantly (*p* < 0.05) lower survival rate (17.49%).

### 3.5. Hemolytic Activity and Susceptibility to Antibiotics

Probiotic strains intended for use in the food industry should meet safety requirements apart from potential health-promoting effects. 

Since the hemolytic activity of LAB cultures has been reported in previous studies [49,59], the type of hemolysis of isolated *L. rhamnosus* strains was investigated. According to our results, no clearing zone on blood agar was observed in any of the wild-type *L. rhamnosus* cultures (Figure S2). Hence, all strains were considered as γ-hemolytic.

Antibiotics resistance (AR) in the *Lactobacillus* genus is under continuous review, and many studies have investigated the resistant phenotype linked with genetic alterations [60,61]. The acquired resistance of LAB and the possible horizontal transfer of the resistance to the gut pathogens or human commensal bacteria are of major concern, and within this context, microbiological cut-off values intend to distinguish susceptible from resistant strains [31]. 

In our study, MIC values of the five *L. rhamnosus* isolates for common antibiotics were determined, and according to the results, all strains were susceptible to all tested antibiotics, apart from chloramphenicol (Table 3). Despite the phenotypical resistance to chloramphenicol, the resulting MIC value (8 mg/L) was just higher than the recommended cut-off value for *L. rhamnosus* species (4 mg/L) [31]. Thus, it is highly unlikely that the resistance was acquired, as microbial growth would be expected even after exposure to a much higher concentration [62]. Similar results were reported for *L. rhamnosus* strains of human and food origin, although for some antibiotics a wide range of MIC values has been presented [63,64]. For example, the MIC values for clindamycin and erythromycin ranged from 0.032 to 8 and 0.016 to 32 mg/L, respectively, and for chloramphenicol from 0.5 to 8 mg/L [63], in accordance with the present study. *L. rhamnosus* L-455 strain displayed high resistance to erythromycin, streptomycin, and clindamycin, despite the absence of resistance genes [63]. Another study determined the MIC values of *L. rhamnosus* strains [64] and presented comparable outcomes with our study, as well. Briefly, MIC values of ampicillin ranged from 0.5 to 8 mg/L by agar dilution and broth microdilution methods, and thus, a new cut-off value of 8 mg/L was proposed. Some strains were characterized as highly resistant to clindamycin, erythromycin, streptomycin, and tetracycline because the corresponding MIC values were higher (i.e., ≥256 mg/L of streptomycin compared with 32 mg/L, which is the cut-off value) than the breakpoints suggested by EFSA. A microarray method suggested the presence of resistance genes; however, it was not confirmed by PCR reactions [64].

In line with all mentioned above, a recent phylogenetic analysis revealed that within the *L. casei-manihotivorans* group, over 80% of the studied strains displayed chloramphenicol resistance, which was associated with the *cat* gene coding for chloramphenicol acetyltransferase [60].

Undoubtedly, antibiotic resistance is an issue under continued discussion that may be affected by many factors, such as the strain’s origin, applied methods, etc. Hence, future research is needed to gain insight into the safe use of probiotics or starter cultures in food production.

## 4. Conclusions

In summary, the results presented in the present work suggest the great potential of the newly isolated wild-type *L. rhamnosus* strains in maintaining gut homeostasis and as antidiabetic agents to alleviate T1D symptoms. However, further confirmation of their efficiency in experimental animal models is considered an essential next research step.

## Figures and Tables

**Table 1:** Basic characteristics of wild-type *L. rhamnosus* strains and α-glucosidase inhibition (%).

Isolate Code	Bacterial Species	Source of Isolation	CFS pH	CFS α-Glucosidase Inhibition (%)
CHTH-2	*Lacticaseibacillus rhamnosus*	Feta-type cheese	3.76 ± 0.01	35.43 ± 2.57 ^d^
OLXAL-1	*Lacticaseibacillus rhamnosus*	Olive (fruit)	3.76 ± 0.04	44.87 ± 0.88 ^c^
OLXAL-2	*Lacticaseibacillus rhamnosus*	Olive (fruit)	3.72 ± 0.02	41.33 ± 0.49 ^a–c^
OLXAL-3	*Lacticaseibacillus rhamnosus*	Olive (fruit)	3.72 ± 0.01	37.73 ± 1.63 ^ad^
OLXAL-4	*Lacticaseibacillus rhamnosus*	Olive (fruit)	3.75 ± 0.02	42.52 ± 2.31 ^bc^
GG (ATCC 53103)	*Lacticaseibacillus rhamnosus*	Human intestines	3.87 ± 0.01	39.68 ± 0.03 ^ab^

The values with different letters in superscript differ significantly (*p* < 0.05). CFS: cell-free supernatant.

**Table 2:** Tolerance of LAB strains to simulated digestion phases.

Strains	Survival Rate (%)		
	Simulated Oral Phase	Simulated Gastric Phase	Simulated Intestinal Phase
*L. rhamnosus* GG	99.74 ± 0.67	77.12 ± 0.32 ^ab^	0
*L. rhamnosus* OLXAL-1	99.79 ± 0.59	75.77 ± 1.46 ^a^	36.46 ± 1.49 ^bc^
*L. rhamnosus* OLXAL-2	99.64 ± 0.51	74.29 ± 1.59 ^ab^	33.07 ± 1.39 ^a^
*L. rhamnosus* OLXAL-3	99.95 ± 0.70	76.54 ± 1.09 ^a^	36.76 ± 0.95 ^c^
*L. rhamnosus* OLXAL-4	99.64 ± 0.22	73.26 ± 0.70 ^b^	34.27 ± 0.05 ^ab^
*L. rhamnosus* CHTH-2	99.45 ± 0.07	76.24 ± 0.95 ^a^	17.49 ± 1.13 ^d^

Significant differences (*p* < 0.05) are shown with letters in superscript.

**Table 3:** MICs values (mg/L) determined for new wild-type *L. rhamnosus* strains in LAB susceptibility test medium (LSM) broth by the microdilution method [31].

Isolate	T	K	G	S	E	A	Ch	Cl
OLXAL-1	1	64	8	8	1	4	4	1
OLXAL-2	1	64	16	8	1	8 ^R^	8 ^R^	1
OLXAL-3	1	64	16	16	1	8 ^R^	8 ^R^	1
OLXAL-4	1	64	16	16	1	4	8 ^R^	1
CHTH-2	1	64	8	16	1	4	8 ^R^	1
Cut-off values	8	64	16	32	1	4	4	1

T: Tetracycline, K: Kanamycin, G: Gentamicin, S: Streptomycin, E: Erythromycin, A: Ampicillin, Ch: Chloramphenicol, Cl: Clindamycin, R: Resistant.

## Data Availability

The data presented in this study are available on request from the corresponding author. The data are not publicly available due to restrictions of the funding authorities.

## Supplementary Materials

The following are available online at: https://www.mdpi.com/article/10.3390/microorganisms10020272/s1, Figure S1: Growth inhibition activity of cell-free supernatants (CFSs) of *L. rhamnosus* strains against food spoilage and pathogenic microorganisms, Figure S2: Hemolytic activity of (a) *L. rhamnosus* OLXAL-1, (b) *L. rhamnosus* GG, and (c,d) *S. aureus* MRSA.
