# Supplementary material for: New Wild-Type Lacticaseibacillus rhamnosus Strains as Candidates to Manage Type 1 Diabetes
Source: Microorganisms. 2022 Jan 25;10(2):272. doi: 10.3390/microorganisms10020272 (PMC8875344; doi:10.3390/microorganisms10020272)
Supplement: Supplementary file 1 [file microorganisms-10-00272-s001.zip › microorganisms-1534535-supplementary.pdf]

Supplementary figures: Figure S1 and Figure S2

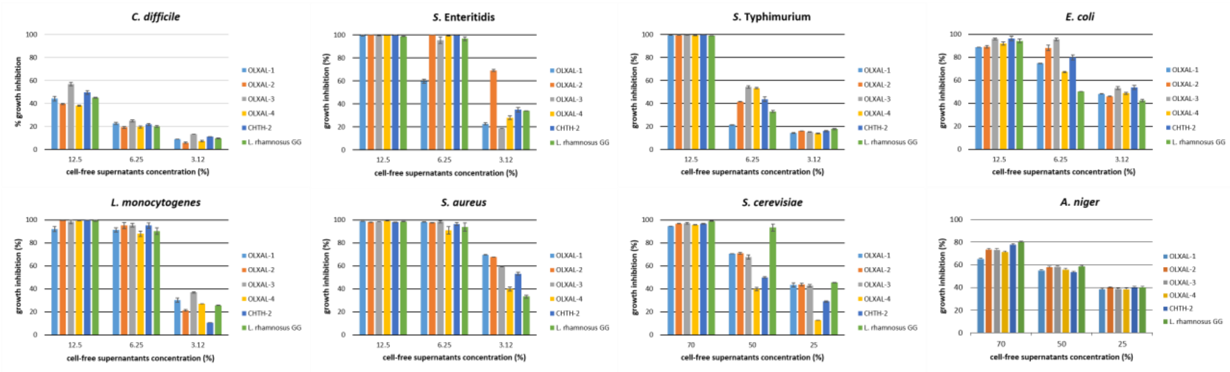

Figure S1. Growth inhibition activity of cell-free supernatants (CFSs) of *L. rhamnosus* strains against food spoilage and pathogenic microorganisms.

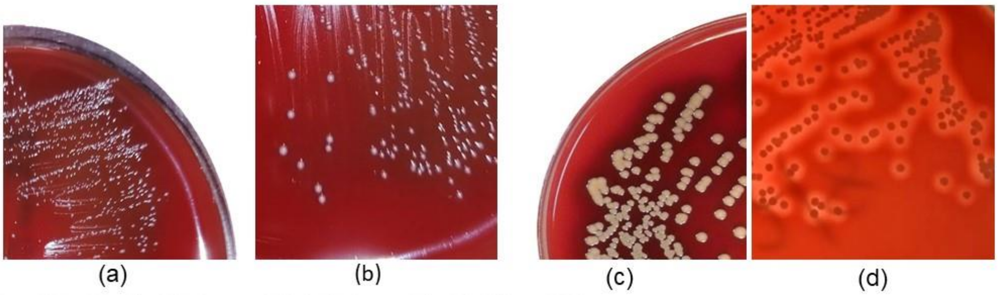

Figure S2. Hemolytic activity of (**a**) *L. rhamnosus* OLXAL-1, (**b**) *L. rhamnosus* GG, and (**c**), (**d**) *S. aureus* MRSA.
